# Deep developmental transcriptome sequencing uncovers numerous new genes and enhances gene annotation in the sponge *Amphimedon queenslandica*

**DOI:** 10.1186/s12864-015-1588-z

**Published:** 2015-05-15

**Authors:** Selene L Fernandez-Valverde, Andrew D Calcino, Bernard M Degnan

**Affiliations:** Centre for Marine Sciences, School of Biological Sciences, The University of Queensland, Brisbane, 4072 Australia

**Keywords:** Transcriptome, Transcription termination, Alternative splicing, Metazoan evolution

## Abstract

**Background:**

The demosponge *Amphimedon queenslandica* is amongst the few early-branching metazoans with an assembled and annotated draft genome, making it an important species in the study of the origin and early evolution of animals. Current gene models in this species are largely based on *in silico* predictions and low coverage expressed sequence tag (EST) evidence.

**Results:**

*Amphimedon queenslandica* protein-coding gene models are improved using deep RNA-Seq data from four developmental stages and CEL-Seq data from 82 developmental samples. Over 86% of previously predicted genes are retained in the new gene models, although 24% have additional exons; there is also a marked increase in the total number of annotated 3’ and 5’ untranslated regions (UTRs). Importantly, these new developmental transcriptome data reveal numerous previously unannotated protein-coding genes in the *Amphimedon* genome, increasing the total gene number by 25%, from 30,060 to 40,122. In general, *Amphimedon* genes have introns that are markedly smaller than those in other animals and most of the alternatively spliced genes in *Amphimedon* undergo intron-retention; exon-skipping is the least common mode of alternative splicing. Finally, in addition to canonical polyadenylation signal sequences, *Amphimedon* genes are enriched in a number of unique AT-rich motifs in their 3’ UTRs.

**Conclusions:**

The inclusion of developmental transcriptome data has substantially improved the structure and composition of protein-coding gene models in *Amphimedon queenslandica*, providing a more accurate and comprehensive set of genes for functional and comparative studies. These improvements reveal the *Amphimedon* genome is comprised of a remarkably high number of tightly packed genes. These genes have small introns and there is pervasive intron retention amongst alternatively spliced transcripts. These aspects of the sponge genome are more similar unicellular opisthokont genomes than to other animal genomes.

**Electronic supplementary material:**

The online version of this article (doi:10.1186/s12864-015-1588-z) contains supplementary material, which is available to authorized users.

## Background

The origin of the fundamental rules governing metazoan multicellularity and morphological complexity can be gleaned through the analysis of the genomes of early branching animals (e.g. sponges, cnidarians, ctenophores and placozoans) [1-4] and their closely related unicellular holozoans (e.g. choanoflagellates and filastereans) [5-7]. Comparative analysis of these genomes has shed light into the evolution of protein-coding gene families. For instance, transcription factor and signalling pathway gene families that are essential to the development of complex bilaterians (e.g. vertebrates, insects, worms and their allies) largely evolved in the Precambrian, before the lineage leading to these animals diverged from early branching animal phyla [1,8-11].

Obtaining a more complete picture of the origin and early evolution of metazoan multicellularity and development also requires the analysis of the mechanisms that regulate gene expression. This demands (i) a more precise view of genome organisation and composition, and gene structure, (ii) detailed expression profiles from multiple cell types, and developmental and physiological contexts, and (iii) the capacity to experimentally manipulate gene function. Thus, increasing the accuracy and completeness of the draft genomes of early branching metazoans is an important step in improving their utility for future evolutionary and functional studies aimed at unravelling the origin of animal multicellularity.

The genome of the demosponge *Amphimedon queenslandica* was published in 2010 [1] and is currently the only published genome from phylum Porifera. The sponge body plan is amongst the simplest in the animal kingdom. It lacks nerve and muscle cells and a centralised gut (reviewed in [1,12-14]). Porifera is traditionally regarded as the oldest surviving phyletic lineage of animals. However, as recent molecular phylogenomic and phylogenetic analyses both support [1,15] and reject [3,4,16,17] this traditional view, it remains unclear as to whether sponges or ctenophores are the sister group to all other animals and whether poriferans are monophyletic. Thus, interpretations of the sponge body plan in the context of metazoan evolution range from it representing a state similar to the last common ancestor of modern animals to it being derived from a morphologically more complex ancestor that possessed a gut, nerves and muscles.

Here we have improved the gene annotations in the draft genome of *Amphimedon* by combining deep transcriptome data from four developmental stages with previously generated developmental ESTs and CEL-Seq – a single cell RNA-Seq method [18] - evidence across 82 sponge developmental samples, from early cleavage through metamorphosis [19]. The inclusion of these transcriptomes markedly improves the current *Amphimedon* protein-coding gene models, which were primarily based on *ab initio* predictions and low-throughput EST evidence, and increases the total number of protein-coding genes in the genome by 25%. Furthermore, analysis of transcripts across sponge development has for the first time revealed alternative splicing patterns in a sponge, which are more similar to those reported in yeast than to those described in eumetazoans.

## Results

### Evidence-based protein-coding gene annotation

**Figure 1:**
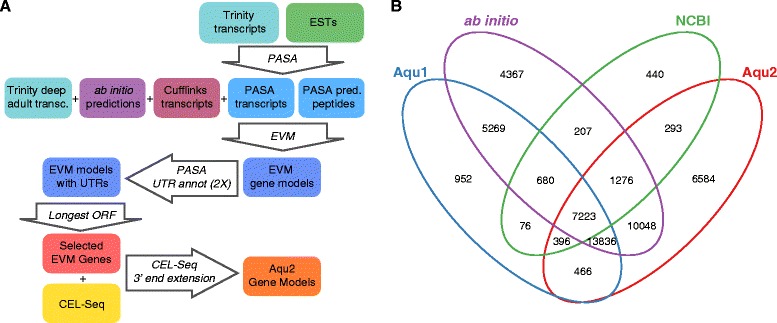
Reannotation strategy and comparison of the new gene models (Aqu2) of the *Amphimedon queenslandica* genome with previous gene models (Aqu1, *ab initio* and NCBI). **A)** Diagram of *de novo* transcriptome assembly and annotation strategy. Boxes represent sets of data while arrows denote specific computational steps in the annotation pipeline. Steps involving Trinity have been omitted for brevity. **B)** Venn diagram showing overlap of Aqu2 models with previous annotations including *ab initio* (Augustus, SNAP and GenomeScan), NCBI and Aqu1 at 80% similarity to account for missing UTR regions in previous annotations. Intersections were done in a hierarchical fashion with the following order of precedence: Aqu2; Aqu1; *ab initio*; and NCBI.

We sequenced and assembled *de novo A. queenslandica* polyadenylated RNAs present in adult, juvenile, competent and pre-competent larval stages in a strand-specific manner using Trinity [20]. To help detect low-abundance transcripts we also sequenced an adult sponge sample at high-depth in an unstranded manner and assembled it *de novo* with Trinity [20] (Table 1, see Methods). All strand-specific transcripts were combined with 8,880 previously assembled EST contigs from larval stages [1] using PASA [21]. The best open reading frames (ORFs) were predicted from the representative transcripts generated by PASA (Figure 1A). To better resolve *A. queenslandica* gene families characterized by complex and highly repetitive regions that Trinity might assemble incorrectly (e.g. the Nucleotide-binding domain and Leucine-rich Repeat- containing (NLR) gene family [22]), an independent genome-guided assembly for each developmental stage was generated using Cufflinks [23]. Only Cufflinks transcripts found in at least two developmental stages were used as additional evidence for gene annotation (Figure 1A).

**Table 1:** **Transcriptome sequencing statistics**

	**Precompetent larvae**	**Competent larvae**	**Juvenile**	**Adult**	**Adult deep**
**Reads**	42,273,865	43,699,007	41,677,487	43,098,690	125,880,671
**Quality trimmed reads**	39,234,338	40,546,526	38,587,047	39,976,627	116,384,700
**Assembled transcripts**	107,725	122,306	101,432	112,187	230,181
**Average transcript length**	816	738	706	716	1,278
**Longest transcript**	14,660	12,052	9,354	16,961	29,513
**Mapped Transcripts**	63,681	64,826	61,149	65,377	76,045
**% Mapped/Assembled**	59.11%	53.00%	60.29%	58.28%	33.04%
**Average mapped transcript**	738	729	673	686	918
**Longest mapped transcript**	14,393	12,052	24,959	16,954	29,166
**Total transcripts mapped to genome**	63,681	64,826	61,149	65,377	76,045
**Total coverage (% genome)**	28.32%	27.92%	29.24%	30.31%	21.62%
**Used for PASA**	Yes	Yes	Yes	Yes	No

*De novo* and genome-based assembled transcripts, predicted ORFs and the previously generated *ab initio* gene models [1] were combined using EVM [24] to predict protein-coding gene models. Untranslated regions (UTRs) were added to these EVM gene models by two successive rounds of PASA using all developmental stranded Trinity transcripts and ESTs (Figure 1A).

The completed set of *Amphimedon* genes - Aqu2 - contains a total of 47,895 transcripts, which includes alternatively spliced gene isoforms expressed in different developmental stages (see below). To reduce isoform redundancy we identified each gene’s isoform with the longest ORF (Figure 1A), resulting in 40,122 protein-coding loci in the final Aqu2 gene annotation*.* Finally, deep 3’ end-biased CEL-Seq [18] expression data spanning 82 *A. queenslandica* developmental samples [19] were used to refine the 3’ ends of Aqu2 gene models, resulting in the extension of the 3’ ends of 10,925 genes (Figure 1A).

### Comparison with previous annotations

Currently, the main *Amphimedon* gene annotation resource available to the community is Aqu1. Aqu1 has 30,060 genes and was released along with the original report of the *Amphimedon* genome [1] (Table 2). Additionally, NCBI generated a limited set of predicted genes via their automated pipeline upon genome submission, resulting in 9,975 protein-coding gene predictions. To assess the gene annotation improvements, Aqu2 was compared with these gene annotations and previously generated *ab initio* gene model predictions [1] (Figure 1B).

**Table 2:** **Aqu1 and Aqu2 gene annotation comparison**

	**Aqu1**	**Aqu2**
**Total gene number**	30,060	40,122
**Total length (% genome)**	69.3 Mb (47.82%)	93.5 Mb (64.52%)
Average gene length *	2,426 bp	2,521 bp
Smallest gene *	81 bp	149 bp
Longest gene *	86,995 bp	103,992 bp
**Total isoform number**	30,060	47,895
**Total exon number**	171,753	206,984
**Average exon length**	218 bp	225 bp
**Average exons per gene**	5.7	5.2
**Max exons per gene**	97	103
**Total intron number**	140,027	166,862
**Average intron length**	253 bp	327 bp
**ORF**		
**Total length**	35.6 Mb	42.6 Mb
**Average size**	1,184 bp	1,062 bp
**Longest**	47,676 bp	56,682 bp
**5’ UTRs**		
**Total number**	4,457	9,873
**Total length**	0.6 Mb	1.13 Mb
**Average size**	130 bp	114 bp
**Longest**	2,318 bp	4,952 bp
**3' UTRs**		
**Total number**	6,021	14,892
**Total length**	1. 3 Mb	2.8 Mb
**Average size**	211 bp	188 bp
**Longest**	2,268 bp	2,814 bp

*Includes introns and exons.

21,921 (54.6%) Aqu2 models share at least 80% identity with Aqu1 models, with many of the revised genes having a different structure (i.e. exon-intron architecture) or length (Figure 1B). 4,340 Aqu1 gene models are not supported at all in the Aqu2 annotation. Also 43,279 (71.6%) of *ab initio* and 7,918 (79.4%) of NCBI annotated genes are included in Aqu2. Some NCBI models are fragmented into smaller gene models resulting in 9,188 Aqu2 models from 7,918 NCBI models (Figure 1B). In contrast, many adjacent *ab initio* models have been merged resulting in 32,383 Aqu2 models from 43,279 *ab initio* models (Figure 1B).

**Figure 2:**
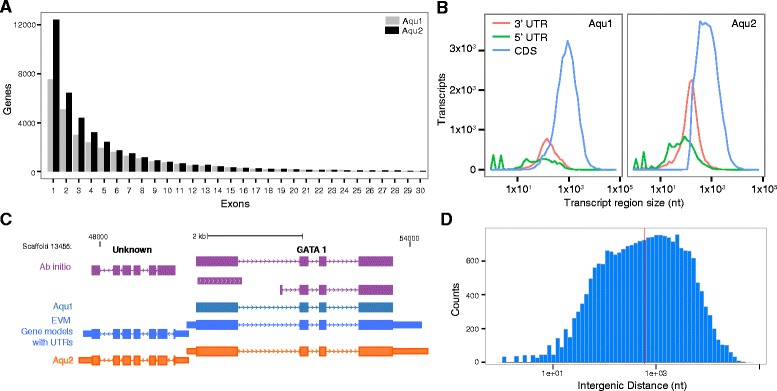
Improvement in *Amphimedon queenslandica* gene annotations. **A)** Increase in the number of exons (x-axis) per gene (y-axis) between Aqu1 (gray) and Aqu2 (black) for all genes with less than 30 exons. **B)** Increase in the number (y-axis) and size of coding (CDS - blue) and non-coding (3’ UTR – pink, 5’ UTR – green) transcript regions (x-axis) between Aqu1 (left panel) and Aqu2 (right panel). The transcript region size is displayed in log-scale. **C)** Genome browser example of improvements in gene annotations between *ab initio* (Augustus, SNAP and GenomeScan – purple track), Aqu1 (dark green-blue track), EVM models with annotated UTRs (bright blue) and Aqu2 (orange track). The gene on the right side of the panel is *GATA* and the one on the left has no significant match in other organisms. Thick blocks represent coding exons, thin blocks non-coding exons and lines introns. Small arrows on introns denote the direction of transcription. Scaffold number and position, and scale bar shown on top. **D)** Distribution of the intergenic distance between annotated genes of *Amphimedon queenslandica* (Aqu2) displayed in log-scale. The median intergenic distance (587 bp) is shown as a vertical line (red).

Aqu2 covers 16.7% more of the current *A. queenslandica* genome (Table 2) and includes 35,231 newly annotated exons, with 5,309 of the previous Aqu1 models having additional exons in their corresponding Aqu2 models (Figure 2A). There is also a marked increase in the number of 3’ and 5’ untranslated regions (UTRs) (Figure 2B and Table 2). The use of CEL-Seq 3’ end evidence results in an increase from 6,021 to 14,892 annotated 3’ UTRs (Table 2) in Aqu2, while 5’ UTRs only increase from 4,457 to 9,873 (Table 2).

A comparison of the protein best blast hit (BBH) of Aqu1 and Aqu2 gene models against the SwissProt database reveals that Aqu1 and Aqu2 have a similar number of BBH to metazoan proteins, with slightly fewer matches in Aqu2 (419 proteins). In contrast, Aqu2 has more unique coding sequences (i.e. no blast matches in the database) with identifiable PFAM domains proteins (3,804) compared than Aqu1 (2,649) (Additional file 1: Figure S1), potentially expanding the list of lineage-restricted genes. Finally, there are 17,310 unannotated genes in Aqu2 compared to 7,879 in Aqu1, which will require further verification to establish if they are present in other basal metazoans, unique to poriferans, or restricted to demosponges.

Improvements in Aqu2 are exemplified in the locus depicted in Figure 2C, which shows the gene encoding the developmental transcription factor GATA [1,25-27], and a previously unannotated gene transcribed in the opposite direction from a putative bidirectional promoter. This gene was missing from the previous annotation (Aqu1) although it was predicted by *ab initio* methods (Figure 2C). In Aqu2 both genes have annotated 3’ and 5’ UTRs; CEL-Seq data further extend both the 3’ ends. The significant increase in gene model number and length results in a more gene dense genome with a decrease of the median intergenic distance from 929 to 587 bp (Figure 2D).

### Identification of previously unannotated protein-coding genes

Although Aqu2 represents a more complete picture of the genes present in the *Amphimedon*, we find most improvements in conserved gene families are minor and are generally restricted to more accurate assignment of exons and untranslated regions. However there are a few notable exceptions. For instance, we identified a number of new transcription factors, including the *Aristaless* homeobox (*ArxC*) gene, which, in spite of having been previously identified by Larroux et al. [28] was missing from the Aqu1 annotation. In this case, the 5’ end of the corresponding Aqu1 model is discarded in Aqu2 and the 3’ end extended. The discarded 5’ end encodes the splicing factor 3B subunit and now comprises the adjacent gene. Aqu2 also includes a new member of the POU transcription factor gene family and previously unannotated genes encoding neuronal proteins including the Synapse Differentiation-Induced Protein1-Like (Capucin), a gene expressed in the caudal and putamen brain regions of mouse and human, and a new version of CPEB, a protein involved in memory maintenance [29-32] (Additional file 1: Figure S2A,B).

### *Amphimedon* possesses both conserved and novel transcription termination elements

**Figure 3:**
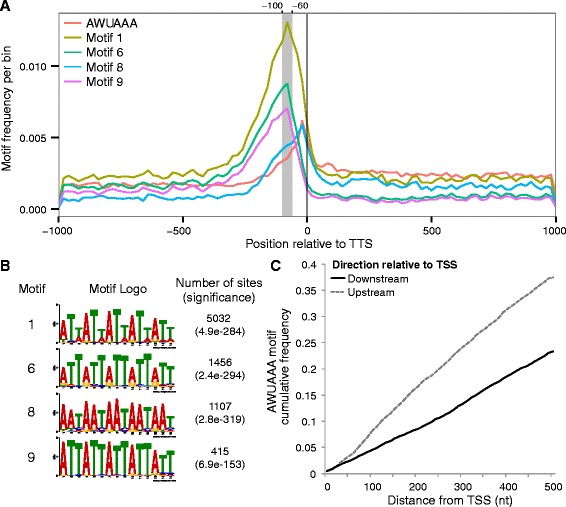
Transcription termination elements overrepresented in *Amphimedon queenslandica* 3’ UTRs. **A)** Frequency per 20 bp bin (y-axis) of enriched motifs in mRNA 3’ ends signals 2,000 bp around the transcription termination site (TTS), including the consensus polyadenylation signal (PAS) AWUAAA (orange line). Motif numbers correspond to those in panel B and relate to the motif ranking found by MEME. The most prevalent position of novel motifs 1, 6 and 9 is highlighted in the grey shaded area (−60 to −100 bp relative to TTS). **B)** Sequence logo, number of occurrences and significance for four AT-rich motifs enriched before the canonical polyadenylation signal (PAS). The significance value is the e-value of the log likelihood ratio of each motif, while the number of sites per class shows the number of 3’ UTRs out of a total of 10,274 strict 3’ UTRs that had a particular motif. **C)** Cumulative frequency of the PAS consensus sequence (AWUAAA) 500 bp upstream (dashed grey line) or downstream (solid black line) of a set of strict transcription start sites (TSS) on scaffolds longer than 50 kb and not overlapping with other TSSs.

We identified motifs enriched in 10,274 strict 3’ UTRs that are now annotated in *Amphimedon*. There are four long AT-rich motifs that are overrepresented in this region, three of which sit between 100 and 60 bp upstream of the transcription termination site (TTS) (Figure 3A,B). These motifs are more abundant than the polyadenylation signal sequence (PAS) consensus sequence (AWUAAA), which is found adjacent to and preceding the TTS (Figure 3A). One of the identified motifs - motif 8 - is a composite version of the polyA signal (AATx5 – Figure 3B) that, as expected, overlaps with the canonical PAS sequence (Figure 3A).

Comparison of the cumulative frequency of the consensus PAS relative to the transcription start site (TSS) reveals PASs accumulate more rapidly upstream than downstream of a set of 3,309 strict TSSs (Figure 3C). This pattern of a lower frequency of PASs on the coding strand is consistent with PAS being associated with transcription termination in *A. queenslandica* [33].

### Alternative splicing is dominated by intron retention

A conservative estimate of alternative splicing (AS) in *A. queenslandica* was obtained by considering only AS events supported by at least three different assembled transcripts (Additional file 1: Figure S3). Only AS events resulting in the acquisition of an alternative first or last exon were lowly supported, with 98% of these appearing, at most, in two different assembled transcripts (Additional file 1: Figure S3).

**Figure 4:**
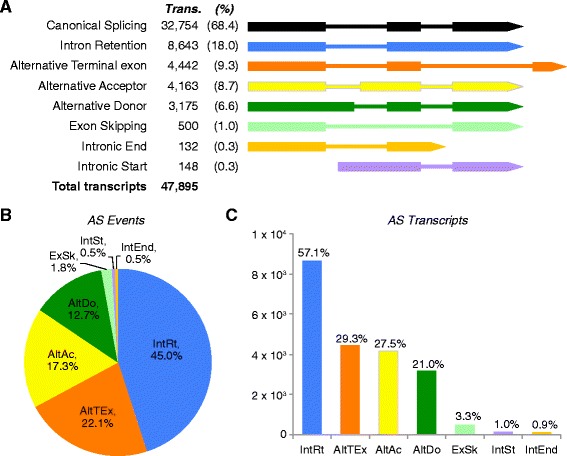
Alternative splicing in *Amphimedon queenslandica*. **A)** Graphical depiction of AS event classes in relation to the canonically spliced isoform (black). From top to bottom: intron retention (IntRt - blue), alternative terminal exon (AltTEx - orange), alternative acceptor (AltAc - yellow), alternative donor (AltDo - green), exon-skipping (ExSk – light green), intron end (IntEnd - light orange) and intron start (IntSt - violet). The number of transcripts in each class of AS is shown in the second column and the percentage of all transcripts this represents is shown in parentheses. The total percentage is over 100% as different types of AS sometimes occur in the same transcript. **B)** Pie chart showing percentage of AS events belonging to the classes shown in panel A. **C)** Number of transcripts (y-axis) and percentage (number above each bars) of transcripts with AS events (x-axis) exemplified in panel A. Similar to panel A, the total percentage is over 100% as different types of AS events can occur in the same transcript. Colour coding and abbreviations in B and C are identical to those used in panel A.

Based on these conservative estimates, alternative splicing in *A. queenslandica* appears to be less prevalent than in many eumetazoans [34], with less than 32% of the total transcripts detected in this study being generated by some form of AS (Figure 4). The large majority of AS events result in the retention of an intron, constituting 45% of all alternative splicing events and 57.1% of all alternatively spliced transcripts (Table 3 and Figure 4). The second most abundant splicing event results in the incorporation of an alternative terminal exon (22.1% of AS events), followed by alternative splice acceptor (17.3%) and donor (12.7%) (Table 3 and Figure 4).

**Table 3:** **Alternative splicing in** ***Amphimedon queenslandica***

**Abbreviation**	**AS Type**	AS events*	**Number of transcripts**	**% of AS events**	**% of AS transcripts** ^**+**^	**% of All transcripts**
**IntRt**	Intron retention	12,610	8,643	45.0%	57.1%	18.0%
**AltTEx**	Alternative Terminal Exon	6,191	4,442	22.1%	29.3%	9.3%
**AltAc**	Alternative Acceptor	4,843	4,163	17.3%	27.5%	8.7%
**AltDo**	Alternative Donor	3,562	3,175	12.7%	21.0%	6.6%
**ExSk**	Exon skipping	513	500	1.8%	3.3%	1.0%
**IntSt**	Intronic Start	148	148	0.5%	1.0%	0.3%
**IntEnd**	Intronic End	132	132	0.5%	0.9%	0.3%

*AS events supported by three or more transcripts.

^**+**^The total percentage of transcripts is higher than 100% as different types of AS events can occur in the same transcript.

## Discussion

The use of high-coverage developmental transcriptomes has markedly improved the gene annotations in the *Amphimedon queenslandica* genome, resulting in the refinement of existing gene models and the identification of a large number of previously unannotated genes. Given the high density of genes in the *Amphimedon* genome, the use of stranded RNA-Seq was proved essential for accurate gene identification and gene model prediction. The integration of CEL-Seq data from 82 developmental samples, spanning from early cleavage through metamorphosis into the juvenile form, further improves the gene models by (i) allowing 3’ UTRs to be extended to regions that have CEL-Seq sequence support and (ii) confirming the developmental expression of new gene models.

Combining stranded-RNA Seq and CEL-Seq data with existing gene models via a pipeline that relies both on *de novo* and genome-informed assemblies significantly improves the accuracy of existing gene models. Improvements include the addition or extension of 3’ and 5’ UTRs, the identification of missing exons, the removal of incorrectly predicted exons and the refinement of exon/intron boundaries. The high level of transcriptome coverage identified genes not included in previous annotations. This approach also has allowed us to identify gene models that were previously fused or fragmented in Aqu1. Although Aqu2 is primarily based on transcriptome evidence, both Aqu1 and Aqu2 show similar coverage of metazoan orthologues and support the expression of conserved metazoan proteins during *Amphimedon* development.

A number of biological observations emerge from these new gene annotations. First, the increase in the number of protein-coding genes in the *Amphimedon* genome has led to the expansion of some gene families, including those encoding developmental transcription factors, such as Arx and POU, and proteins involved in neuron functioning, such as Capucin and CPEB. It is worth noting that although the Aqu2 models have led to a better annotation of metazoan gene families, most conserved gene families were accurately annotated in Aqu1.

Second, the more accurate annotation of untranslated regions in Aqu2 allows for the identification to transcription start and termination sites. Genomic sequences in the vicinity of these sites contribute to the regulation of gene transcription and transcript termination and stability. Core transcriptional elements overlap with TSSs [35-38] and PASs and other motifs in the 3’ UTR control transcript termination, stability and localisation [39,40]. Analysis of the 3’ UTRs in *Amphimedon* reveals the enrichment of a number of AT-rich motifs 60–100 bp upstream of TTS. These currently appear to be unique to *Amphimedon*. Further, as observed in vertebrates [33], analysis of the frequency of PAS sequences upstream and downstream of putative TSSs in *Amphimedon* reveals a disproportionate depletion of PASs in the direction of transcription compared to in the opposite non-coding direction. This is consistent with PAS signals participating in transcription termination in *Amphimedon*.

Third, the extension of existing genes and the annotation of new genes both have contributed to an overall increase in gene density. Indeed, the *Amphimedon* genome is the most gene dense animal genome currently known [5]. In addition to having minimal intergenic spacing (median = 0.59 kb), intron size in *Amphimedon* is markedly smaller than other animals (see [5] and Table 2). Both intergenic and intron size in *Amphimedon* are more similar to non-metazoan opisthokonts [1,5,23]. Given the basal position of poriferans, these characteristics suggest sponges may have retained genomic features of the first metazoans. Although protein-coding gene content in sponges includes many metazoan innovations [1,5,9-11], their genome organisation and gene structure appears to be more similar to simple unicellular ophistokonts.

Fourth and consistent with the above observation, the level and modes of alternative splicing in *Amphimedon* is more akin to those found in yeast than in other animals. This sponge has lower proportion of alternative splicing events compared to other animals, particularly those that result in exon-skipping and gene product diversification. These very low levels of exon-skipping are similar to those observed in yeast [41,42] and in contrast to bilaterians, where exon-skipping is often the most prevalent form of AS [42,43]. As an increase in average intron size correlates with increased levels of exon-skipping [34,44], the limited exon-skipping and small intron size in *Amphimedon* are consistent with these genomic features and processes emerging later in eumetazoan evolution, after the divergence of this and the sponge lineage.

Fifth, the new Aqu2 models greatly expand the number of gene models without orthologues to over 20,000. Nearly all these genes are developmentally expressed based on RNA-Seq and CEL-Seq data. With a paucity of whole genome data from phylum Porifera it is currently difficult to reconstruct the evolutionary history of these genes.

## Conclusions

In improving the accuracy of the *Amphimedon* gene models we have increased the number of full-length genes with accurate transcription start and termination sites. This allows for the future identification and analysis of promoters and other regulatory sequences populating intergenic DNA and UTRs. Combined with experimental manipulation and a detailed analysis of gene expression, the analysis of *cis*-regulatory DNA provides a means to understand the logic underlying sponge morphogenesis and cell specification and differentiation. When placed in a comparative framework, this knowledge informs our understanding of the evolution of the cell types [24,45] and developmental mechanisms underpinning metazoan body plans.

## Methods

### Sample collection and sequencing

Adult, juvenile, and competent and pre-competent larvae of *Amphimedon queenslandica* sponges were collected from Heron Island Reef, Great Barrier Reef, Queensland, Australia as previously described [46]. Total RNA from each developmental stage was extracted using the standard TRIzol regent protocol (Invitrogen) and genomic DNA was removed by DNase treatment. The RNA quality was assessed using the Agilent 2100 Bioanalyzer. RNA was paired-end sequenced using the Illumina HiSeq2000 platform (Illumina, San Diego). All samples were sequenced in a strand sensitive fashion. We additionally sequenced an adult sponge tissue sample at high-depth in an unstranded manner to help detect low-abundance transcripts (Table 1).

### De novo transcriptome assembly

Raw paired-end sequences were quality filtered using Trimmomatic [47]. The first 7 bp of each read were cropped and reads were subsequently trimmed if the average quality within a window of 4 bp was below 15. Unpaired reads and reads smaller than 60 bp were discarded. Quality-filtered paired-end reads were assembled de novo using Trinity [20] (Table 1). Each developmental stage was assembled independently with default parameters, with the exception of a lower transcript size cut-off of 200 nt and jaccard-clipping. These assembled transcripts for each developmental stage were aligned and condensed using the PASA pipeline [24], where only transcripts with more than 90% transcript coverage (parameter --MIN_PERCENT_ALIGNED) and 95% identity (parameter --MIN_AVG_PER_ID) to the genome were merged. Peptides were predicted from these transcripts using Transdecoder [48] and used as further evidence for gene annotation (see below).

High-coverage unstranded adult sponge reads were quality checked and trimmed as described above. Remaining reads were independently assembled *de novo* three times using Trinity with default parameters, using a lower transcript size cut-off of 200 nt and jaccard-clipping, but including a minimum kmer coverage of 2, 4 and 10 (−−min_kmer_cov parameter). The three Trinity assemblies were subsequently merged using CAP3 [49] at 95% similarity and a minimum overlap of 100 bp. These sequences were mapped to the genome using gmap with a minimum of 90% identity [50] and provided as further transcript evidence to EVM [24], but not included in the main PASA transcript set (see Figure 1A, Table 1).

### Reference based transcriptome assembly

Quality filtered reads from the four stranded libraries (Table 1) were mapped to the *A. queenslandica* genome [1] using Tophat2 [51] and assembled using Cufflinks2 [23]. Each developmental stage was assembled separately and shared transcripts were collapsed using Cuffmerge [23]. The gtf file obtained by Cuffmerge was converted to gff3 format and used as further evidence for gene annotation.

### Evidenced based gene prediction and UTR annotation

Gene evidence and predicted gene structure were combined using EVM [24]. The evidence included a) *ab initio* predictions generated by Augustus, SNAP and GenomeScan [1], b) PASA generated consensus transcript assemblies based on both the stranded developmental Trinity assemblies and publically available Sanger ESTs [1], c) the Transdecoder predicted peptides of PASA consensus transcripts, d) the high-depth adult transcriptome and e) the genome-guided gene models generated by Cufflinks. RNA-Seq evidence was strongly favoured over *ab initio* and other predictions using the weight system incorporated in EVM. The evidence weights are summarized in Table S1. UTRs were added onto the gene models predicted by EVM [24] by two sequential PASA [24] rounds including annotation loading, annotation comparison and annotation updates to maximize incorporation onto gene models predicted by EVM, as per the suggestion of the authors in the PASA pipeline manual (http://pasapipeline.github.io/) (see Figure 1A).

### CEL-Seq gene 3’ end extension

CEL-Seq developmental data (stages: cleavage, brown, cloud, spot, late spot, ring, late ring and swimming larvae) for *Amphimedon* developmental stages were retrieved from NCBI’s Gene Expression Omnibus (GSE54364) and are described in detail in [19]. 24 additional samples spanning from post-settlement postlarvae undergoing metamorphosis into the juvenile form and adult were provided by the Yanai lab (Anavy et al. unpublished). CEL-Seq reads were processed, quality filtered and mapped back to the *A. queenslandica* genome (ampQue1) using BWA [51] through the CEL-Seq analysis pipeline [52]. To identify transcript ends, we clustered all overlapping reads mapped to the same DNA strand in each individual developmental sample. Developmental stage replicates were processed individually. Clusters with at least 10 reads were retained. Clustered regions were identified in several developmental samples in a stranded fashion; resulting in a total of 74,973 CEL-Seq based clusters. We extended the 3' end of all EVM gene models with annotated 3’ UTRs whose last annotated exon had at least 10 bp overlap with these CEL-Seq clusters, only if their annotated 3' end was shorter than the one supported by CEL-Seq (Figure 1A).

### Gene annotation

Open reading frames (ORFs) for all genes were predicted using Transdecoder [48]. All best ORF candidates were analysed for protein domains, signal sequences and transmembrane domain using hmmer 3.0 [53], signalp 4.1 [54], blastp + [55] and tmhmm 2.0 [56], and combined to annotate each ORF using Trinotate ([24]). Novel candidate genes were manually verified as related to other known genes in nr, RefSeq and SwissProt databases using the web interfaces of Blast and PSI-blast [55]. Their protein domains were also verified using the web versions of SMART [57] and InterProScan [58]. The sequences of newly annotated proteins discussed in the text are provided in the Additional file 1: Supplementary material. The complete set of predicted peptides and gene annotations can be accessed at http://amphimedon.qcloud.qcif.edu.au/index.html.

### Assembly comparison

The assembly comparison shown in Figure 1B was done by intersecting Aqu1, *ab initio* gene models (Augustus, SNAP and GenomeScan), NCBI and Aqu2 annotations. An 80% genome coverage threshold was used to account for missing UTR regions in previous annotations. As in some cases a single Aqu1 gene might correspond to two or more Aqu2 genes or vice versa, we have used a hierarchical approach using the following order of precedence: Aqu2; Aqu1; *ab initio*; and NCBI. Only the original reference (Aqu2) set will be identical in number in the Venn diagram as the original number of elements in the set, while all other comparison sets (Aqu1, *ab initio* and NCBI) will have more or less elements depending on their overall correspondence with the reference set. Other intersections, such as the number of Aqu1 genes that are not supported in Aqu2, as well as the number of *ab initio* and NCBI covered in Aqu2 were done using overlapSelect (parameters: −overlapThreshold = 0.8 –strand) from the UCSC toolkit [59].

### 3’ end motif identification

10,274 3’ UTR sequences overlapping with CEL-seq based clusters found on genomic scaffolds longer than 50 kb were searched for nucleotide motifs using MEME (parameters: −maxsize 20000000 -p 14 -dna -nmotifs 10 -minw 6 -maxw 15 -mod zoops) [60]. We restricted our search to sequences in scaffolds longer than 50 kb to avoid PAS signal depletion due to lack of adjacent sequence. Motif frequency matrices were converted from MEME to Homer format and used to map their frequency around the annotated TTS and TSS using the Homer toolkit [61]. For the cumulative PAS signal distribution analysis, strict TSSs were defined as those of genes found in scaffolds longer than 50 kb whose promoters (100 bp upstream and 50 bp downstream of the TSS) did not overlap with other genes.

### Alternative splicing analysis

The four stranded developmental transcriptomes and EST data were combined using the PASA pipeline with the alternative splicing detection option [24]. AS events supported by less than three transcripts were considered as lowly supported and removed from subsequent analyses.

### Availability of supporting data

The transcriptome sequencing data has been submitted to NCBI’s Sequence Read Archive (SRA) with accession number SUB596470. The new gene annotations, gene and transcriptome nucleotide and peptide sequences can be downloaded from our website (http://amphimedon.qcloud.qcif.edu.au/downloads.html). The new Aqu2 annotations can be visualized at our local genome browser (http://amphimedon.qcloud.qcif.edu.au/genome_browser.html).

CEL-Seq data can be access through the Gene Expression Omnibus (GEO) with GEO Accession GSE54364 [56].

## Additional file

Additional file 1:
**Supplemental material including: Examples of novel proteins in Aqu2.** **Figure S1.** Blastp best blast hit (BBH) annotation comparison. **Figure S2.** Improvements to the annotation of CPEB proteins. **Figure S3.** Transcript support for alternatively splicing events. **Table S1.** Weight of transcript evidence used for gene prediction via EVM.

## Abbreviations

EST: Expressed sequence tag
AS: Alternative splicing
TSS: Transcription start site
TTS: Transcription termination site
AltTEx: Alternative terminal exon
IntRt: Intron retention
AltAc: Alternative acceptor
AltDo: Alternative donor
ExSk: Exon-skipping
IntEnd: Intron end
IntSt: Intron start
UTR: Untranslated region
nt: nucleotide
bp: base pair
Mb: Megabase
