# Supplementary material for: Deep developmental transcriptome sequencing uncovers numerous new genes and enhances gene annotation in the sponge Amphimedon queenslandica
Source: BMC Genomics. 2015 May 15;16(1):387. doi: 10.1186/s12864-015-1588-z (PMC4432959; doi:10.1186/s12864-015-1588-z)
Supplement: Additional file 1: — Supplemental material including: Examples of novel proteins in Aqu2. Figure S1. Blastp best blast hit (BBH) annotation comparison. Figure S2. Improvements to the annotation of CPEB proteins. Figure S3. Transcript support for alternatively splicing events. Table S1. Weight of transcript evidence used for gene prediction via EVM. [file 12864_2015_1588_MOESM1_ESM.docx]

**Supplemental Material**

**Deep developmental transcriptome sequencing uncovers numerous new genes and enhances gene annotation in the sponge *Amphimedon queenslandica***

Selene L. Fernandez-Valverde, Andrew D. Calcino and Bernard M. Degnan^*^

Centre for Marine Sciences, School of Biological Sciences, The University of Queensland, Brisbane 4072, Australia

* Corresponding author

**Email addresses:**

Selene L. Fernandez-Valverde – [uqslizbe@uq.edu.au](mailto:uqslizbe@uq.edu.au)

Andrew D. Calcino – a.calcino@uq.edu.au

Bernard M. Degnan – [b.degnan@uq.edu.au](mailto:b.degnan@uq.edu.au)

**Corresponding author contact details:**

Bernard M. Degnan

School of Biological Sciences

University of Queensland

Brisbane QLD 4072, Australia

Phone: +61 7 336 52467

E-mail: [b.degnan@uq.edu.au](mailto:b.degnan@uq.edu.au)

**Supplemental Data**

**Examples of novel proteins in Aqu2.**

>AmqArxc_Refined - Aqu2.30094_001

MQNSSVLIVHPSDYRFPMATGNPAGKFGGRHYSLDSPGSLTSPPAYDMPLMPGAGPEQKPGVSALSMQNSCAGGQMSMSSCSPPFSQINPLVYSHPGHAGLLPLNSTSLATVHAPNSHAGMYLHHPSAGPMVPSSMSIGRSEPYRKGKQRRHRTNFTSHQLEELEKAFEKTRYPDVFMREELAMKISLTEARVQVWFQNRRAKWRKAEKAAAAANQKDSKDSEDQESPVSSPAPSDGKGASSPKKSTSSPLSSPPARKMHPSTGGNESWTSSPVDSFSPPTFHSPPQSPSIIPTSCPTPNPIHSYTPESPFAAMGIVSHQTPTGTGAPTGAGHFTNSYLQSMTRYAPHC*

>Amq_POU_New - Aqu2.15915_001

MNNHPSRPPAASSAPEEWPPDFSAQHQFHYQYQNLQHGQPETIPLPSQQQPSAYSTDFNLDSFLAADLNAMYAAQAAATAGGRSQEYLQPLSVPLHYQVGHSHHSIDLEQQRPHSHSIVSSGHYVPQVPSRPLSEGLHTSITPPLRPSCRYQVHYDRGEGASFQRAGTQTEQHQQHQFISHCQQYPQHPLNIHHHDHHHCSHVQPAAPTNNPCPLHPNNQLQPLYTSHQHPPVPQQQLSQQQLPQQPLPQQQLPQQQLPQQPVPQQQPSSSNQVLLYHQPPPVHSHLLPQQQLPQQRLPQQQLPQQPLVRRPLPQYHQSLVRQSEYIQPVMAKPTHIIFPPIIHELQKKDSSGSKQVLIPVPLPRTNNDNNSKDSPDLPTAPIPTLPPSSLPSLPSTPYIPEPILSQPPTAPTPVPGPSGYNPDSPDSPIRASCRNVDIEGIRRLRLFADRFKESRIRYEYSKQHVAQQISIRYNFEMTEQQLQQFESKALSFEEMSAMKTHLEKWLMDTLRNRGINETEIKQLSQWLTSFHQRRRRRTAIPVQTKKQLLKEFENNPKPSVKALKALAEKLGIRFEVVRVWFCNKRAKKKAGKDTGPEDDEEIEEELDSDDEGNSDVQSSQ*

>Amq_CPEB1 - Aqu2.37586_001

MEASLGFQTRPLAAVVGKEKCSVSTASGLWSSSVNNSSSSSDMAFSNSLSYALENSLKLWQPTYNGPPSSSLKADWTYTRQSNNTESSCFGAALDYSWSLEYSKETSSPSTLSISSASRTPSPSIAAATSSHPPPSFGTPSSSSSTDVYSRKVFVGGLPPDIDEDEIRDHFIQFGALTVDWPHKAQSKAYFPPKGYAFIIFQEESSVHQLIKICHLDDGKLYMFVSSVTQTNKKVQIRPWKLSDSNYIMDHTQPIDPRKTIFIGGVPRPLKAAELADIFNSRYGNVCYAGIDCDPDLKYPKGAGRVTFSSRVSFMSAVSCRFFQVTYGDIDKKVEVKPYVLDDQICDECRGVNCDGQFAPFFCGNIHCLRYYCEHCWISVHSAPGTQNHRCFIKENGDRPRAINFFSHFPNPSPRPVV*

>Amq_CPEB2 - Aqu2.37587_001

MSGTKDLNGLSRSVVFPDSPSEDMNSLFKDSWSGESNPSPSPRLPFNYSLEDSKSVLTEPACWAYRPLPRLGLQPSSTTFSDCEKTMIPTSSQSHHQLMPPPSITTHRSVSFSDQHGSEYGSPSHGLKYRERVSSLPTAPHHSSLQEQMADHQFQLVDDIFPNVTPEPIETPITQYQTMIGAEAATISKKDKASSRGWLEVSIDCTMPTSPVLRKDTFNFGSLQDTLPSSPTSKSMLQKRVNISPPASGTAASIAIASPVDSNCLVKKLLPLPTDETYSRKVFIGGLPPDIDQEEIRMSFQKYGSVTIDWPHKIHSKSRVPPKGYAFLLFKDEGSVHKLLACCVTENDKLFAYIKYYVTGTSITRKKCQIRPWKITDSYYFANEDKKVDNRKAVFVGGVPRPLKARELAEVMTEKFGPVSFVAIDCDTELKYPKGAACVVFATHASYIASVSSRFMQLVFGALEKKVEVKPYILEDQLCDECNGIQSDGQPAPFFCGSVTCLKYYCEHCWATFHSLSGRQNHRPMFKDIFDRDKFS*

>Amq_CPEB3 - Aqu2.21747_001

MAYTEGMFSQLMEEKQDPVKEKKMSDSSNSSSSSSSSPVEGQETGQTPWTIGLVECPILNSSSSESSIDSGLDSPGAPSSRNSPLSLNDAIVSTSNLTSMLNKLTIAPVSVSDNPFSDSHEFHVPPLSPDYLIPPSPLLKDSLSLSFQSPIPWGASFPGIYQSTHHYGNGPGGRSFEPICSLPYPSSPYSSRPLQSSILSVSAGGSPVCKWSGHLPPRRYKNPTYSPRVFLGGVPWDTTEATLLALFQPFGNLSVDWPGKENKHNRHPPKGYVYLTFEMEKSVKALLMTCGQNPSNAGQYFCKVSSRRIRNKEVQVIPWALSDSNSIRSPSPRLDPSRTVFVGGLHGLINADSLAHIIDELFGGVVYAGIDTDKYKYPIGSGRVTFGNQQGYMKAVKAGFVEIKTPNFTKKVQIDPYLEDNLCSVCGRVVGPYFCRDFQCFKYFCRACWQWQHTTDGFIGHRPLTRTSKSTPHPV*

>Amq_Capucin - Aqu2.13465_001

MGGTSMGGVWCRGTEPGSCHISAVINFKPCILFLFELMAKNLVKDVAEENNYRPLELVEATNASSKDTTSTMITDTGNVLSLQPVFVDERHDDANKFDGRFTFTSADKDLQLYFNSVYQREQSGDAALQNVEDPKDEVVTIFQNKLLSQDADGSYDNSDDRDALAIAPNKDISDSQENSANDNLSVSDRVTPTPTLSDHDSSDGDVIPLSKLNKEELADNNPLLLEVNRLCRNINIGDITHESVILSFNEYFVEFNRQFKQLVCSQNPVQSVICLVVQTAYKLCVDYILEGPVMKTFNDSPHSTINQLPKAVEDILLSRNKLEAIFKPCLINNDDHWELLKTSYSYLLCISKARSLRPFIEIQNQTLAIVSDQIEEPIQKLIEQAVHVTWDMVTLVPPAILAQPPDINEEWHEIRTTYWNDSTHNPLIYFKPVLFYSALGQVACKGEVGNVLRSNAVVLHHQEPETRSQQSSENISHEAYPVPDLSIMLNEADDEISNQVEKNELQLKVFHSVSIKTSPPAKISYAPEYEVAWRSCSNCGANAMISDRSTCEHCNNSAPDYLAWSILTLLCCFWPFSILALVYSMETRHHNASSNYKRASKSSKMAMIYNYFSFFTGILLIIILTM

**Supplemental Figures**

**
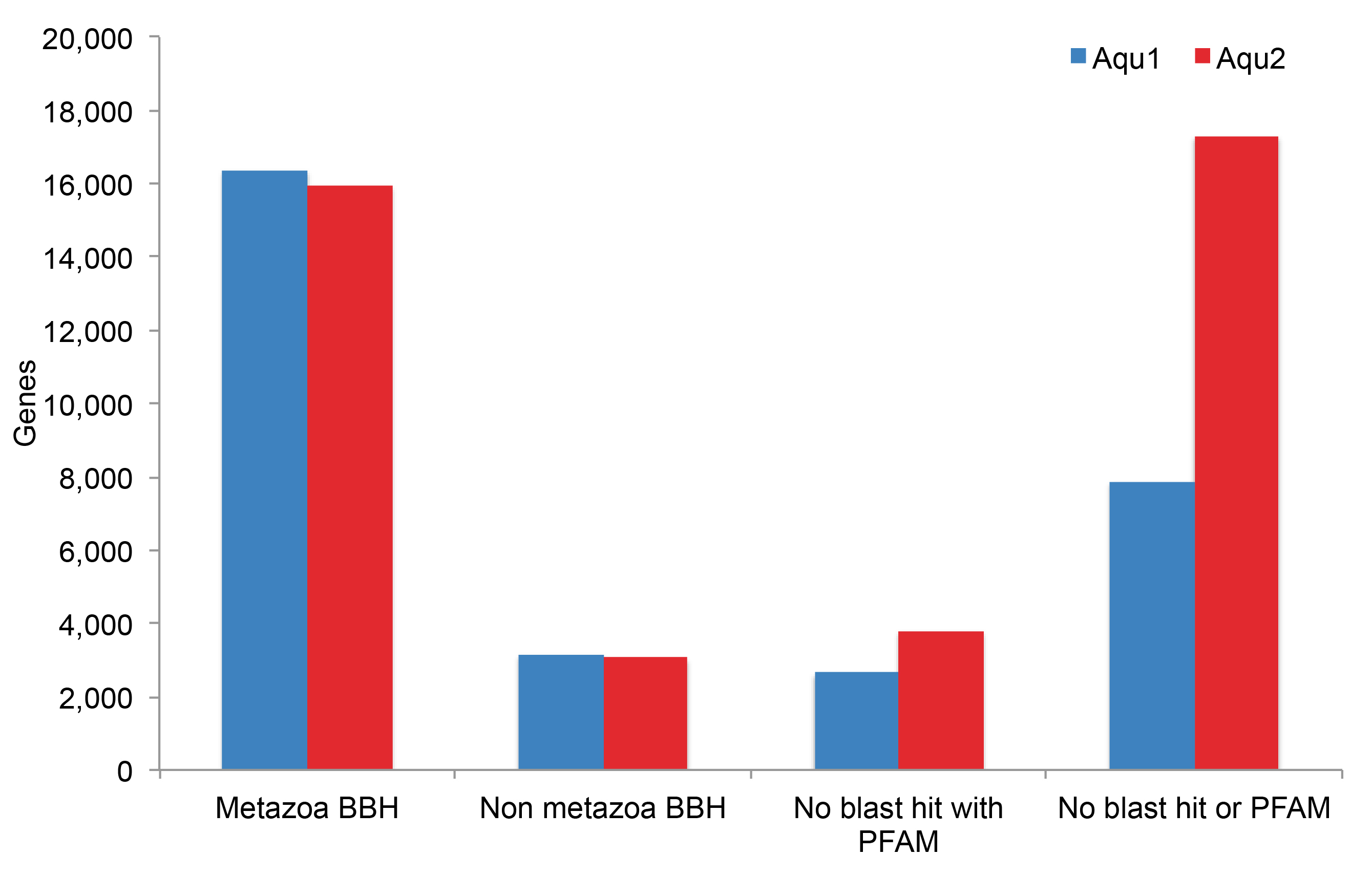
Figure S1- Blastp best blast hit (BBH) annotation comparison.** Number of genes (y-axis) where the BBH is a metazoan or non-metazoan protein. Also shown are proteins that have no significant blast hit yet contain an identifiable PFAM domain, and proteins with no significant blast hit and no identifiable PFAM domain. Aqu1 (blue bars); Aqu2 (red bars).

**
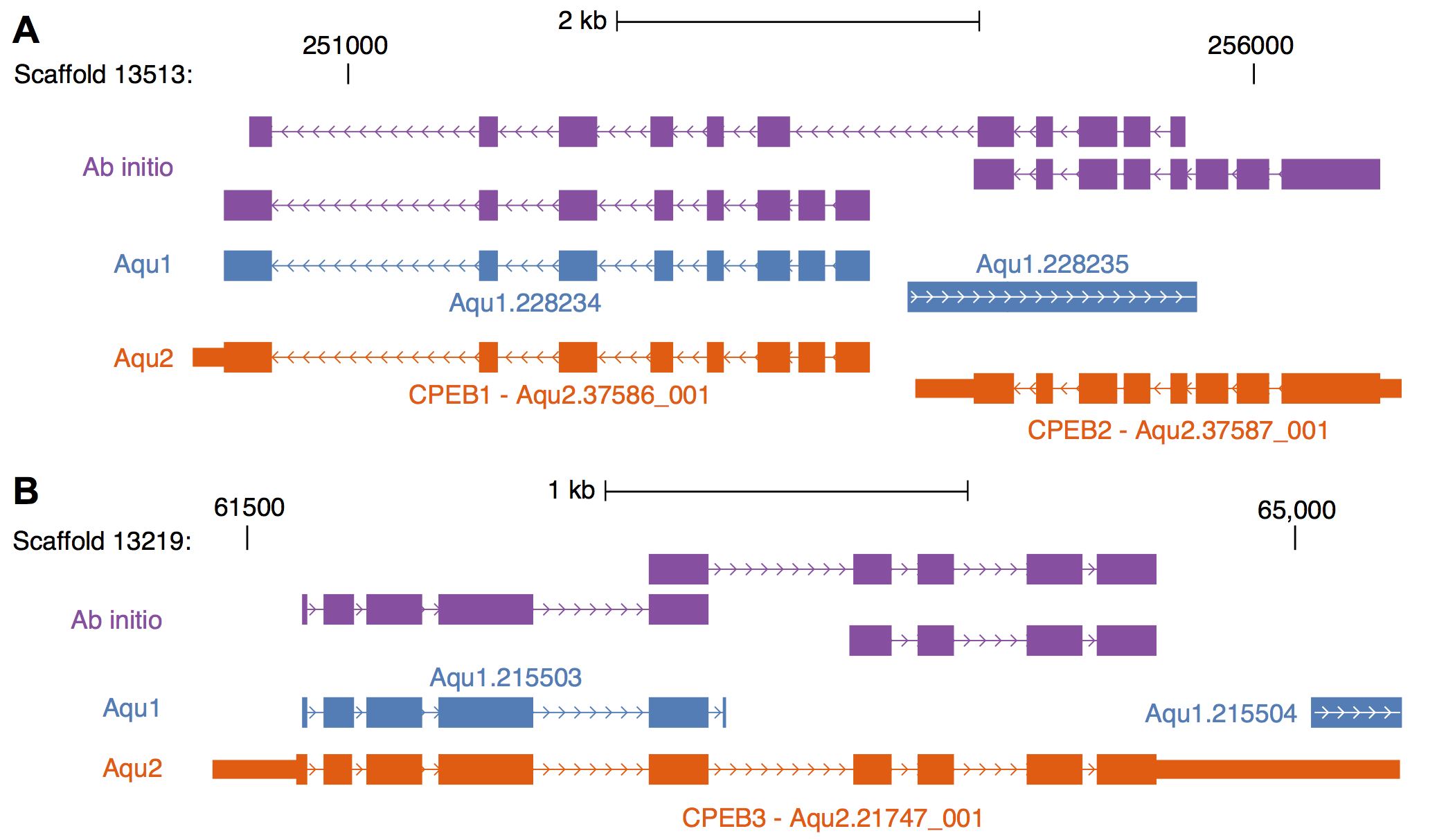
**

Figure S2 – Improvements to the annotation of CPEB proteins. Genome browser example showing *Ab initio* (Augustus, SNAP and GenomeScan – purple track), Aqu1 (dark blue track) and Aqu2 (orange track) gene models. Small arrows on introns or block exons denote the direction of transcription. A) The annotation of CPEB1 was improved via the addition of a 3’ UTR, while a new version of CEBP2 was identified. B) Significant improvements to the annotation of CPEB3 (previously Aqu1.215503), including the addition of four new coding exons and 3’ and 5’ UTRs..


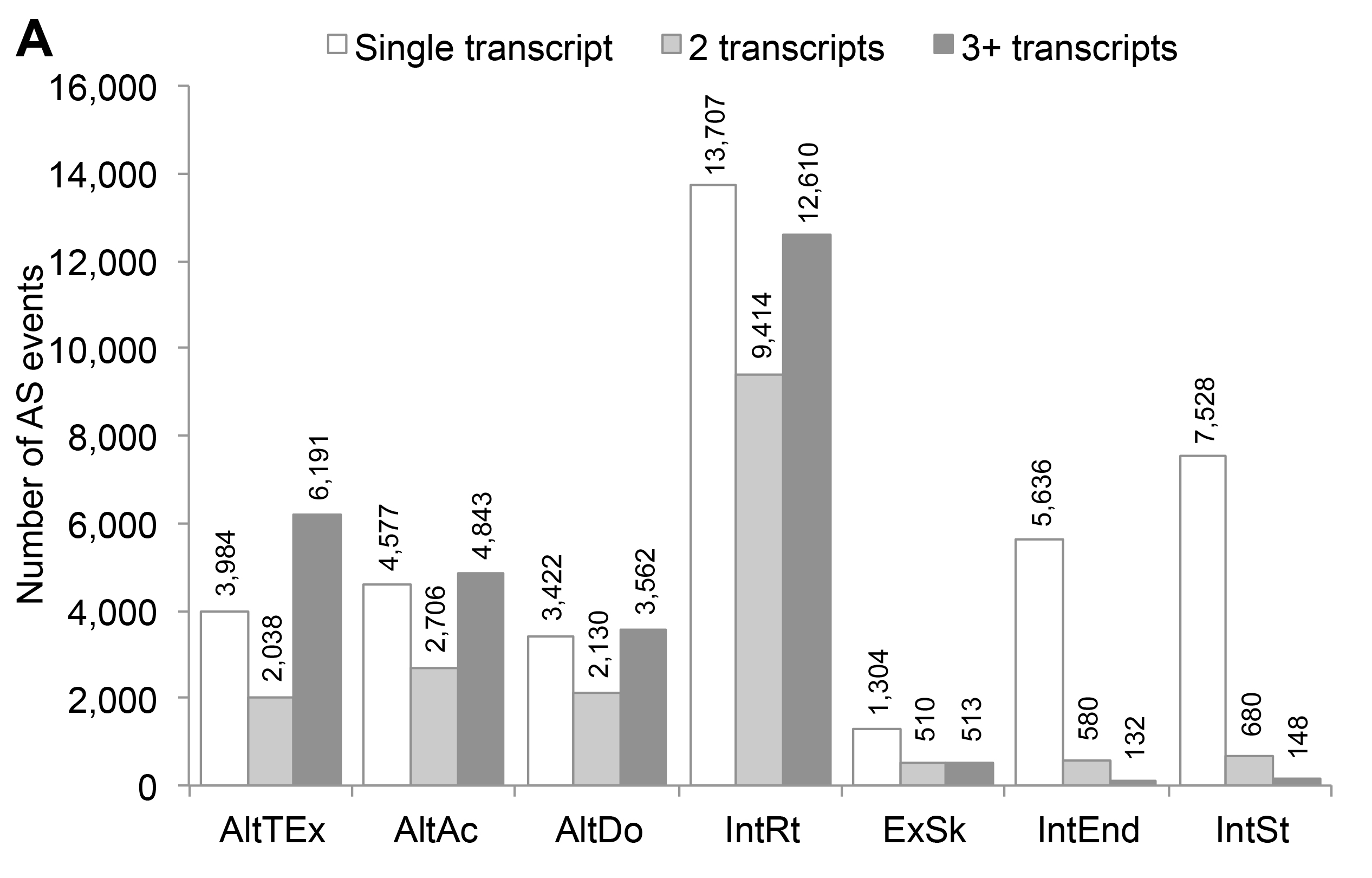


Figure S3 – Transcript support for alternatively splicing events. Each column shows the number (A) of AS events supported by one (white), two (light gray) or three or more (dark gray) transcripts across 4 developmental transcriptomes and a set of ESTs. The AS event classes are (from left to right) alternative terminal exon (AltTEx), intron retention (IntRt), alternative acceptor (AltAc), alternative donor (AltDo), exon-skipping (ExSk), intron end (IntEnd) and intron start (IntSt).

Table S1 – Weight of transcript evidence used for gene prediction via EVM.

| Evidence type | Description | EVM weight |
| --- | --- | --- |
| TRANSCRIPT | Stranded RNA-Seq collapsed by PASA | 10 |
| TRANSCRIPT | High-depth Adult RNA-seq | 3 |
| TRANSCRIPT | Cufflinks assembled transcripts | 4 |
| ABINITIO_PREDICTION | Augustus | 1 |
| ABINITIO_PREDICTION | SNAP | 1 |
| ABINITIO_PREDICTION | GenomeScan | 1 |
| OTHER_PREDICTION | Transdecoder peptides predicted from stranded RNA-Seq collapsed by PASA | 1 |

Note: Weight scale goes from least important (1) to most important (10).
